# Supplementary material for: Feasibility of a virtual reality intervention targeting distress and anxiety symptoms in patients with primary brain tumors: Interim analysis of a phase 2 clinical trial
Source: Res Sq. 2023 Jan 31:rs.3.rs-2522094. Preprint. [Version 1] doi: 10.21203/rs.3.rs-2522094/v1 (PMC9928043; doi:10.21203/rs.3.rs-2522094/v1)
Supplement: 1 [file NIHPPRS2522094V1-supplement-1.pdf]

Secondary aims

1. To assess the effects of the VR intervention on self-reported acute and subacute distress and anxiety symptoms.
2. To determine if the effects VR has on distress and anxiety are more pronounced in those with high distress (based on DT cut-off score of ≥ 5) compared to those with low distress (based on DT cut-off score of < 5).
3. To determine if the effects VR has on distress and anxiety are more pronounced in those individuals not on systemic corticosteroids compared to those who are on active CS therapy.

Exploratory aims

1. To explore the correlations between biological stress measures (as measured by salivary cortisol, dehydroepiandrosterone-sulfate, and alpha amylase with PROs
2. To explore the effects of a VR intervention on PROs collected on the NHS, including mood disturbance, symptom burden and interference, health-related quality of life, and cognitive function
3. To explore the impact of loneliness and financial toxicity on distress and anxiety symptoms during the COVID-19 pandemic
   To determine the proportion of patients with adjustment disorder (AjD) in a PBT population

Supplementary Figure 1. Secondary and exploratory aims of the VR trial. Secondary aims of this trial include assessing the effects of the VR intervention on acute (immediately post-intervention) and subacute (1 to 4 weeks post-intervention) distress and anxiety symptoms while determining if baseline distress levels and corticosteroid use have an impact on intervention efficacy. Exploratory aims assess correlations between salivary stress biomarkers

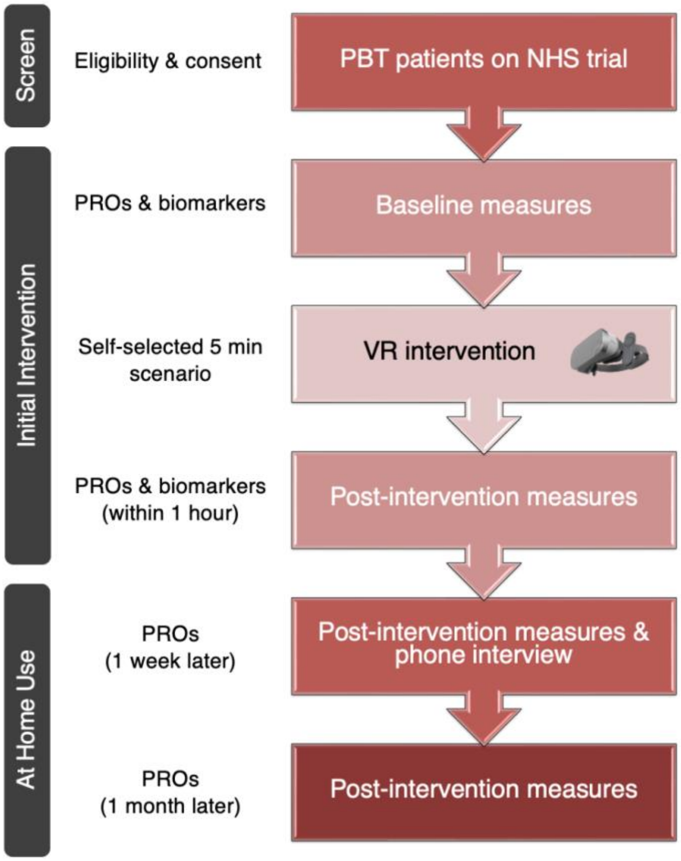

Supplementary Figure 2. Phase 2 trial protocol. Adult PBT patients were recruited from the NOB NHS trial at the NIH. Eligible patients were recruited via email or in clinic and completed baseline PROs and optional salivary stress biomarkers within 2 weeks of their clinical evaluation. Research staff met with patients via telehealth to complete an initial VR intervention where participants self-selected a scenario to complete, followed by post-VR intervention assessments within 1 hour to assess acute effects. Patients then continued VR use at home for the 1 month while on study and repeated post-VR intervention assessments at 1 week and 4

Supplementary Table 1. Responses from Was It Worth It (WIWI) questionnaire

Question	N	Yes (N, %)	No (N, %)
Was it worthwhile for you to participate in the VR intervention?	20	18, 90%	2, 10%
If you had to do it over, would you use VR again?	20	18, 90%	2, 10%
Would you recommend VR to other patients to use before their clinic appointments?	20	19, 95%	1, 5%
Overall, did your quality of life change by using VR?	20	12, 60%	8, 40%
